# Recent advances in understanding and managing T-cell lymphoma

**DOI:** 10.12688/f1000research.12573.1

**Published:** 2017-12-12

**Authors:** Jun Ho Yi, Seok Jin Kim, Won Seog Kim

**Affiliations:** 1 Division of Hematology-Oncology, Department of Medicine, Chung-Ang University , Seoul, Korea, South; 2 Division of Hematology-Oncology, Department of Medicine, Samsung Medical Center, Sungkyunkwan University School of Medicine , Seoul, Korea, South

**Keywords:** T-Cell lymphoma, PTCL, T-lymphocytes, Natural Killer Cells

## Abstract

Owing to the rarity of peripheral T-cell lymphoma (PTCL) and the heterogeneity of subtypes, there are no compelling data to guide the therapeutic approaches for such patients. Over the years, there have been remarkable advances in molecular subtyping and treatment of PTCL, although there are still many areas to be explored. In this review, we summarize recent updates on the evolution of understanding and treatment for PTCL.

## Introduction

T/natural killer (T/NK) cell lymphoma represents a heterogeneous group of malignant lymphoproliferative diseases that arise from T-lymphocytes and NK cells. To distinguish it from immature T-cell neoplasms such as lymphoblastic leukemia/lymphoma, mature T-cell lymphoma is often called peripheral T-cell lymphoma (PTCL). PTCL accounts for approximately 15% of all non-Hodgkin’s lymphoma cases worldwide and shows regional differences in distribution.

Over the years, the molecular understanding of PTCL has advanced remarkably; as a result, the classification of PTCL was recently revised ^1^. In addition, with new combinations of older drugs, novel agents such as checkpoint inhibitors, epigenetic modulators, and anti-folates have been introduced for the treatment of PTCL. Despite this progress, the prognosis for PTCL is worse than that for B-cell lymphoma, even than that before the era of rituximab ^2, 3^.

In this concise review, we provide an overview of the latest advances in the management of PTCL focusing on trials that have been carried out with novel agents. Of note, this review does not cover cutaneous T-cell lymphoma (CTCL) or extranodal NK-T cell lymphoma (ENKTL), because their biological features and treatment strategies differ from those of PTCL.

## Brief review of the 2016 World Health Organization revision of nodal/extranodal peripheral T-cell lymphomas

Beginning with nodal PTCLs, up to 60–100% of angioimmunoblastic T-cell lymphoma (AITL) and up to 40% of PTCL not otherwise specified (PTCL-NOS) demonstrate surface markers of follicular helper T (TFH) cells ^4– 7^ and have common genetic features, such as *RHOA*, *TET2*, *DNMT3A*, and *IDH* ^8– 11^. Two provisional entities have been introduced: follicular T-cell lymphoma and nodal peripheral T-cell lymphoma with the TFH phenotype. These entities can be diagnosed when their neoplastic cells express at least two or three TFH markers but do not display the clinicopathologic features of AITL, which would have been diagnosed as PTCL-NOS according to the 2008 World Health Organization classification. AITL and these two new entities are now categorized as nodal T-cell lymphoma with the TFH phenotype.

By definition, the PTCL-NOS designation encompasses all PTCLs lacking specific features that would allow categorization within any of the better-defined subtypes of PTCL, resulting in heterogeneity of this entity. A recent gene expression profiling study demonstrated that PTCL-NOS can be classified into two molecular groups according to the overexpression of either *GATA3* or *TBX21* ^12^, and the *GATA3* subset shows inferior outcomes. However, as this study has not been applied in routine practice, the results have not led to the description of new entities.

ALK-negative anaplastic large-cell lymphoma (ALCL), which was a provisional entity in the 2008 classifications, has become a definite entity. This CD30-expressing neoplasm is not distinguishable from its ALK-positive counterpart on morphologic grounds, except for the absence of ALK expression. While the prognosis of ALK-negative ALCL is known to be less favorable ^13^, the five-year survival rate is comparable to that of ALK-positive ALC when *DUSP22* rearrangements are found ^14^. After first being described in 1997 ^15^, breast implant-associated ALCL emerged as a distinct clinicopathologic entity, and it was proposed as a new entity in the 2016 classifications. All reported cases are ALK-negative, and the tumor is more frequently confined to the fibrous capsule. These cases show indolent clinical courses and respond well to implant removal and resection of the tumor. However, when the tumor presents with a mass discernible by radiologic or gross pathologic examination, it might be associated with a more aggressive clinical course ^16, 17^.

Major changes in extranodal PTCL cases have emerged from diseases that occur in the gastrointestinal (GI) tract. Enteropathy-associated T-cell lymphoma (EATL) is composed of two subtypes; type I EATL usually occurs following long-standing celiac disease (CD), showing large pleomorphic cells within an inflammatory background, and type II EATL occurs without antecedent CD, showing small, monotonous cells with epitheliotropism. In the 2016 classifications, diagnosis of EATL is to be used only for what was formerly type I EATL. Type II EATL has changed its name to monomorphic epitheliotropic intestinal T-cell lymphoma; it has been proposed as a new entity on the basis of its histologic, genetic, and molecular differences ^18– 20^. Whereas these two highly aggressive diseases show transmural growth, which often leads to GI bleeding or perforation, there is another one that grows superficially along the GI mucosa. Indolent T-cell lymphoproliferative disorder of the GI tract is a new provisional entity that usually presents with chronic diarrhea, weight loss, and malnutrition, mimicking the symptoms of inflammatory bowel disease ^21^. The course of this disease is known to be indolent, although some cases of transformation have been reported ^22^.

## Current standard of care: conventional chemotherapy

Anthracycline-containing regimens such as a combination of cyclophosphamide, doxorubicin, vincristine, and prednisone (CHOP) or CHOP plus etoposide (CHOEP) are recommended as the front-line treatments for PTCLs ^23, 24^. Although clinical outcomes for these regimens vary according to histologic subtype, complete response (CR) rates range from 30% to 70%, and five-year overall survival (OS) rates range from 20% to 60%. Except in several small subsets, such as patients with ALK-positive ALCL or those with a low international prognostic index, the use of anthracycline-containing regimens failed to improve clinical outcomes ^25– 27^. Owing to the lack of efficacy of conventional CHOP, more intensive anthracycline-based regimens have been tried ^28– 32^; however, given limitations of small numbers of patients, intractable toxicities, and unfavorable results, none is considered a standard option. CHOP-14 or CHOEP can still be considered in select patients ^33, 34^.

Various combinations of several non-anthracycline drugs have been evaluated in front-line settings. In the S0350 trial, a combination of cisplatin, etoposide, gemcitabine, and solu-medrol (PEGS) was tried in 26 patients with newly diagnosed PTCL ^35^. The two-year progression-free survival (PFS) rate was 14%, and the two-year OS rate was 36%, which seemed to be no better than those of conventional combinations. Gemcitabine and etoposide were added to CHOP in 26 patients with newly diagnosed PTCL and this resulted in a CR rate of 62% and a median 215 days of event-free survival ^36^. An Italian group reported outcomes of biweekly administration of six cycles of gemcitabine, ifosfamide, and oxaliplatin in 21 patients with high-risk PTCL. The CR rate was 67%, and the five-year event-free survival rate was 49%. In a recent report from the randomized phase II study of the UK group using the combination of gemcitabine, cisplatin, and methylprednisolone (GEM-P), objective response rates (ORRs) were 57.1% for the CHOP arm and 43.2% for the experimental arm ^37^. Although grade 3–4 neutropenia or febrile neutropenia was more common in the CHOP arm, there was no difference in the two-year OS rate (53.1% versus 64.7%) or PFS rate (36.0% versus 39.0%). Outcomes of the recent trials for front-line treatment are summarized in Table 1.

**Table 1.** Summary of the recent trials of front-line treatment for peripheral T-cell lymphoma.

Regimens	Phase	Total accrual	CR/PR rates	Survival outcomes	Grade 3–4 toxicities
CHOP-based combinations					
Everolimus + CHOP ^123^	II	30	57%/33%	2-year PFS: 33% 2-year OS: 70%	Neutropenia: 80% Thrombocytopenia: 60%
Bortezomib + CHOP ^114^	II	46	65%/9%	2-year PFS: 37% 2-year OS: 52%	Neutropenia: 41%
Romidepsin + CHOP ^65^	Ib/II	37	51%/17%	30-months PFS: 41% 30-months OS: 71%	Neutropenia: 89% Thrombocytopenia: 78%
Belinostat + CHOP ^100^	Ib	23	72%/17%	Not reported	Neutropenia: 26% Anemia: 22%
Non-anthracycline combinations					
Cyclophosphamide, etoposide, vincristine, prednisone alternating with pralatrexate ^80^	II	33	52%/18%	2-year PFS: 39% 2-year OS: 60%	
Gemcitabine, etoposide, cisplatin, methylprednisolone ^35^	II	26	23%/15%	2-year PFS: 14% 2-year OS: 36%	Neutropenia: 45% Anemia: 24%
Gemcitabine, cisplatin, methylprednisolone ^37^	II	44	43%/not reported	2-year PFS: 39% 2-year OS: 65%	
Gemcitabine, cisplatin, prednisone, thalidomide ^111^	II	52	52%/15%	2-year PFS: 57% 2-year OS: 71%	Myelosuppression: 44%

CHOP, cyclophosphamide, doxorubicin, vincristine, and prednisone; CR, complete response; OS, overall survival; PFS, progression-free survival; PR, partial response.

Collectively, although there is limited evidence that CHOP or its variants improve the prognosis of patients with PTCL, there is also little evidence that a certain non-anthracycline combination can replace CHOP. A randomized phase II study is under way to compare a combination of ifosfamide, carboplatin, and etoposide (ICE) and dexamethasone with CHOP (NCT02445404).

## Current standard of care: consolidation therapy

Except for ALK-positive ALCLs, which show favorable outcomes with chemotherapy alone, current guidelines recommend consolidative autologous stem cell transplantation (ASCT) in patients with chemosensitive PTCL ^23, 24^. However, this recommendation was not established based on prospective randomized trials.

In a report by Reimer *et al*., 83 patients with PTCL received four to six cycles of CHOP, and for patients in partial response (PR) or CR at the end of induction, ASCT was performed ^38^. Fifty-five patients advanced to ASCT, and the three-year OS for patients who underwent ASCT was 71%. In the NLG-T-01 trial, 115 out of 160 patients with PTCL underwent ASCT after induction treatment of CHOEP-14 or CHOP-14 ^39^. The 5-year PFS and OS rates were 44% and 51%, respectively, and the recently announced follow-up data revealed that the 10-year PFS and OS rates were 38% and 41%, respectively ^40^. Although it appears that prolonged survival can be achieved by ASCT, it should be noted that approximately 30% of patients are unable to receive it and this is mostly due to early progression. Several studies have compared the efficacy of front-line allogeneic stem cell transplantation (alloSCT) with ASCT ^41, 42^; however, the outcomes of alloSCT were not significantly different from those of ASCT. Taken together, these data suggest that ASCT should be considered as the first treatment in patients with chemosensitive PTCL.

## Current standard of care: relapsed or refractory peripheral T-cell lymphoma

Up to 20–30% of patients fail to achieve an initial response, and even after ASCT, over 50% will experience relapsed disease ^38, 39^. For those who have relapsed or refractory PTCL (rrPTCL), salvage chemotherapy with or without subsequent consolidation treatment can produce long-term remission. However, owing to refractoriness of the disease, overall outcomes remain poor ^43^. Several novel agents are actively being investigated in patients with rrPTCL. The major clinical findings of the studies with novel agents are summarized in Table 2.

**Table 2.** Selected studies of novel agents in the treatment of relapsed/refractory peripheral T-cell lymphomas.

Agent	Phase	Total accrual	CR/PR rates	Survival outcomes	Major grade 3–4 toxicities
Monoclonal antibodies					
Brentuximab vedotin	II ^52^	58 (ALCL)	57%/29%	1-year OS rate: 70% 4-year OS rate: 64% 5-year OS rate: 60%	Peripheral sensory neuropathy, neutropenia, thrombocytopenia
	II ^58^	35 (non-ALCL)	24%/18%	Median PFS: 2.6 months	Peripheral sensory neuropathy, neutropenia, hyperkalemia
Mogamulizumab	II ^64^	38	14%/19%	Median PFS: 3.0 months	Lymphocytopenia, neutropenia
Anti-folates					
Pralatrexate	II ^76^	111	11%/18%	Median PFS: 3.5 months Median OS: 14.5 months	Neutropenia, thrombocytopenia, mucositis
Histone deacetylase inhibitors					
Romidepsin	II ^89^	131	15%/11%	Median PFS: 4.0 months Median OS: 11.3 months	Neutropenia, thrombocytopenia
Romidepsin + gemcitabine	II ^94^	20	15%/15%	2-year PFS: 11% 2-year OS: 50%	Neutropenia, thrombocytopenia, anemia
Belinostat	II ^98^	120	11%/15%	Median PFS: 1.6 months Median OS: 7.9 months	Neutropenia, fatigue
Chidamide	II ^102^	83	14%/15%	Median PFS: 2.1 months Median OS: 21.4 months	Neutropenia, thrombocytopenia
Immunomodulatory drugs					
Lenalidomide	II ^108^	54	11%/11%	Median PFS: 1.9 months	Neutropenia, thrombocytopenia, pneumonia, gastrointestinal disorder
	II ^109^	40	8%/18%	Median PFS: 4 months Median OS: 12 months	Neutropenia, pain, dyspnea
Inhibitors of PI3K/mTOR pathways					
Duvelisib	I ^117^	15	13%/33%	Median OS: 36.4 weeks	Hepatitis, rash, neutropenia
Copanlisib	II ^119^	17	14%/7%	Not reported	Hypertension, neutropenia, hyperglycemia
Everolimus	II ^124^	16	6%/38%	Median PFS: 4.1 months Median OS: 10.2 months	Neutropenia, thrombocytopenia, anemia, hyperglycemia
Alternative agents					
Bendamustine	II ^125^	60	28%/22%	Median PFS: 3.6 months Median OS: 6.2 months	Neutropenia, thrombocytopenia, infection
Bendamustine + carboplatin + dexamethasone	II ^126^	30	30%/25%	Median PFS: 4.8 months	Neutropenia, thrombocytopenia, anemia
Alisertib	III ^130^	120	16%/17%	Median PFS: 3.7 months Median OS: 9.9 months	Neutropenia, thrombocytopenia, anemia
Tipifarnib	II ^132^	18	0%/17%	Not reported	Neutropenia, thrombocytopenia

ALCL, anaplastic large-cell lymphoma; CR, complete response; OS, overall survival; PFS, progression-free survival; PR, partial response.

## Monoclonal antibodies

### Alemtuzumab

Alemtuzumab is a humanized monoclonal antibody (mAb) against CD52. As a salvage treatment, it has been evaluated either as monotherapy ^44^ or as combination therapy with various backbones ^45– 47^. Although high CR rates were observed (36–54%), severe opportunistic infections caused concerns. Combinations of alemtuzumab with conventional three-weekly CHOP ^48^, two-weekly CHOP ^49^, four-weekly CHOP ^50^, and CHOEP-14 ^51^ have been assessed in patients with newly diagnosed PTCL. Again, high CR rates (59–71%) were accompanied by profound hematologic toxicity and opportunistic infection. Two international phase III trials are ongoing to evaluate the role of alemtuzumab when added to the first four (out of six) cycles of CHOP-14 (NCT00646854 and NCT00725231).

### Brentuximab vedotin

Brentuximab vedotin (BV) is an antibody-drug conjugate comprising an anti-CD30 mAb conjugated to an anti-microtubule agent, monomethyl auristatin E. As CD30 is typically expressed in Hodgkin’s lymphoma and ALCL, most major findings have been achieved in those diseases. In a pivotal phase II trial, patients with relapsed or refractory systemic ALCL received BV 1.8 mg/kg every three weeks for up to 16 cycles ^52^. Among 58 patients, the ORR was 86% (n = 50) and 33 patients (57%) achieved CR. The four- and five-year survival data demonstrated that long-term remission can be achieved by BV treatment ^53, 54^. For PTCLs other than ALCL, the rate of CD30 positivity varies according to subtype and report ^14, 55– 57^. During a phase II trial in which 35 patients with relapsed or refractory CD30-positive PTCL (22 PTCL-NOS and 13 AITL) received BV at a dose of 1.8 mg/kg every three weeks, the ORR was 41% (95% confidence interval 24.6–59.3), and the PFS of patients with AITL was longer than that of the patients with PTCL-NOS (6.7 versus 1.6 months) ^58^. Several BV-based combinations have also been evaluated. In a phase I trial, 39 treatment-naïve patients with a diagnosis of CD30-positive PTCL were recruited to receive either sequential treatment (two cycles of BV, six cycles of CHOP, and eight cycles of BV) or combination treatment (six cycles of BV in combination with vincristine-omitted CHP and 10 cycles of BV) ^59^. There were seven patients with non-ALCL PTCL—two PTCL-NOS, two AITL, two adult T-cell leukemia/lymphoma (ATLL), and one EATL—they were all allocated to a combination arm for which a CR rate of 100% was noted. A subsequent four-year follow-up analysis revealed that six out of seven of them were still alive ^60^. Based on these favorable outcomes, a randomized phase III trial is ongoing to compare BV-CHP versus CHOP in patients with CD30-positive PTCL (ECHELON-2 (A Comparison of Brentuximab Vedotin and CHP With Standard-of-care CHOP in the Treatment of Patients With CD30-positive Mature T-cell Lymphomas)) trial, NCT01777152).

### Mogamulizumab

Mogamulizumab is a humanized mAb targeting the CC chemokine receptor 4 (CCR4), which is expressed in physiologic regulatory T cells. CCR4 is also expressed in nearly 90% of ATLL cases ^61^ and in approximately 30–65% of patients with PTCLs ^62, 63^, and expression of CCR4 is associated with poor survival. In a Japanese phase II trial, 29 patients with CCR4-positive, relapsed PTCL were recruited to receive mogamulizumab at a dose of 1.0 mg/kg per week for eight weeks ^64^. Five CRs and PRs, which made up 34% of the ORR, were noted. Lymphopenia was the most frequent adverse event (grade 3–4, 73%), and 51% of patients experienced skin disorders of any grade. The degree of CCR4 expression was not correlated with the clinical response. However, in a European phase II trial, a lower ORR was noted ^65^. Of 35 patients with rrPTCL, only one CR and three PRs were noted, and the ORR was 11.4%. The lower ORR can be explained by the different setting (relapsed only versus relapsed or refractory patients), the poorer performance status of the European study population (0% versus 40% of patients were Eastern Cooperative Oncology Group performance status 2), or the lower dose intensity of the administration schedule (weekly administration for eight weeks versus weekly administration for four weeks followed by biweekly administration).

### Immune checkpoint inhibitors

It has long been recognized that the tumor microenvironment plays important roles in lymphomagenesis, proliferation, and immune evasion ^66, 67^. In T-cell lymphoma, the microenvironment defines the tumor itself (such as in AITL), and many subtypes manifest strong tissue tropism (such as in CTCL or primary GI lymphomas).

Given that PD-1 is expressed in a substantial portion of PTCL cases ^68^ and that PD-L1 is frequently expressed in certain virus-associated lymphomas ^69^, this pathway is of major interest. Nivolumab is a fully human anti-PD1 mAb. In a phase I, dose-escalating study, a total of 81 patients with lymphoid malignancy (B-cell lymphoma 31, T-cell lymphoma 23, and multiple myeloma 27) received nivolumab at doses of 1 or 3 mg/kg every two weeks ^70^. Among five patients with PTCL, two PRs were observed, and the median PFS was 14 weeks. In the two responding patients with PTCL, a sustained duration of response (DoR) was observed. Several trials are ongoing to evaluate the role of this agent in patients with PTCL as a monotherapy (NCT03075553 and NCT02973113) or as combination therapy with BV (NCT0258163). Evaluation of pembrolizumab, another PD-1 mAb, in PTCL has been based on anecdote ^71, 72^, and two studies are under way for patients with rrPTCL (NCT03021057 and NCT02362997). In regard to mAbs targeting PD-L1 or PD-L2 or both, prospective studies with avelumab (NCT03046953) and durvalumab (NCT03161223 and NCT03011814) are taking place.

Ipilimumab is a fully humanized mAb targeting cytotoxic T lymphocyte-associated protein 4 (CTLA-4). In a phase I study, nivolumab combined with ipilimumab was given at 3 mg/kg or 1 mg/kg every three weeks for four doses, and this was followed by nivolumab monotherapy every two weeks ^73^. Eleven patients with heavily pre-treated T-cell lymphoma were included, one PR and four standard deviations were noted, and one patient proceeded to alloSCT.

## Anti-folates

Anti-folates demonstrate anti-tumor efficacy by inhibiting dihydrofolate reductase, which converts dihydrofolate to tetrahydrofolate. Depletion of tetrahydrofolate disrupts the synthesis of pyrimidines and amino acids such as serine, glycine, and methionine ^74^.

### Pralatrexate

In an early phase trial with pralatrexate, a higher CR was observed in patients with PTCL compared with those of B-cell lymphoma at the cost of significant mucositis ^75^. This toxicity was later found to be alleviated by the administration of vitamin B _12_ and folic acid.

In the PROPEL (Pralatrexate in Patients with Relapsed or Refractory Peripheral T-Cell Lymphoma) trial, a total of 111 patients with rrPTCL received pralatrexate at a dose of 30 mg/m ^2^ for six out of seven weeks along with vitamin B _12_ and folic acid ^76^. Among 109 evaluable patients, 12 CRs (11%) and 20 PRs (18%) were noted. The median duration of response and the median OS were 10.1 and 14.5 months, respectively. The most common grade 3–4 toxicities were thrombocytopenia (32%), mucositis (22%), neutropenia (22%), and anemia (18%), but pralatrexate was well tolerated, as the overall dose intensity was 80%. In the Japanese trial with the same dose and schedule, nine (45%) out of 20 evaluable patients achieved an objective response, and grade 3–4 thrombocytopenia occurred in 40% of cases ^77^.

Based on the synergism pralatrexate has shown in preclinical analyses ^78, 79^, several combinations with it have been evaluated. In a phase II trial, a front-line combination of cyclophosphamide, etoposide, vincristine, and prednisone (CEOP) alternating with pralatrexate was evaluated in 33 patients with untreated PTCL ^80^. Seventeen CRs (52%) and six PRs (18%) were achieved, and 15 patients received ASCT. The two-year PFS and OS rates were 39% and 60%, respectively, similar to outcomes with CHOP. Grade 3–4 anemia (27%), febrile neutropenia (18%), mucositis (18%), and thrombocytopenia (12%) occurred. In a phase I trial, pralatrexate followed by gemcitabine was administered in 34 patients with relapsed or refractory lymphoproliferative malignancies, among whom 11 patients with PTCL were included ^81^. Of the 33 patients who were evaluable for response, seven (21%) showed a partial response, and two of the seven had T-cell lymphoma. In another case series, five elderly patients with rrPTCL received weekly administration of pralatrexate (15 mg/m ^2^) and bortezomib (1.3 mg/m ^2^) for three out of four weeks until progression ^82^. One patient achieved CR after four cycles, which lasted over 12 months.

Several pralatrexate-based combinations, including CHOP (NCT02594267), pembrolizumab plus decitabine (NCT03240211), romidepsin (NCT01947140), and durvalumab (NCT03161223), are under evaluation.

## Histone deacetylase inhibitors

Histone deacetylase (HDAC) inhibitors demonstrate anti-tumor efficacy by upregulating the expression of genes for cell cycle regulators, cell type-specific differentiation, and pro-apoptotic proteins ^83, 84^. Various classes of HDACs with clinical significance are expressed in patients with PTCL ^85, 86^. Two agents have been approved by the US Food and Drug Administration for treatment of PTCL: romidepsin (2011) and belinostat (2014).

### Romidepsin

Romidepsin is a cyclic tetrapeptide-derived class-I selective HDAC inhibitor. After favorable outcomes were observed in an early phase trial ^87^, a phase II trial was carried out in 47 patients with rrPTCL ^88^. Romidepsin was administered in three out of four weeks with a starting dose of 14 mg/m ^2^, which could be escalated up to 17.5 mg/m ^2^ in the absence of toxicity. Among 45 evaluable patients, eight (18%) experienced CR and nine (20%) experienced PR. Thrombocytopenia was observed in 47% of patients; of note, Epstein-Barr virus (EBV)-associated lymphoproliferative disorder emerged in two patients. In the pivotal phase II trial ^89^, a total of 131 patients with rrPTCL received romidepsin at a dose of 14 mg/m ^2^ for three out of four weeks. Among 130 evaluable patients, 19 (15%) experienced CR and 14 (10%) experienced PR. Rapid responses were observed, and median time to response was 1.8 months and median time to CR was 3.7 months. In the 19 patients who achieved CR, the median PFS was 18 months. Grade 3–4 thrombocytopenia and neutropenia occurred in 23% and 18% of cases, respectively. The updated efficacy data demonstrated that, of the 19 patients who achieved CR, 10 had long-term (at least 12 months) responses ^90^. In the phase II part of the Japanese trial, 40 patients with rrPTCL received 14 mg/m ^2^ of romidepsin ^91^. The CR rate was 25% (10/40), and the PR rate was 18% (7/40). Treatment-related adverse events led to discontinuation of romidepsin in 26% of patients.

Interestingly, an *in vivo* and *in vitro* study has suggested a role for romidepsin in the treatment of EBV-associated cancer, where it can induce the EBV lytic cycle ^92^. In a Korean pilot study, however, three out of five patients with NK/T-cell lymphoma experienced EBV reactivation after romidepsin treatment ^93^. This finding is consistent with the findings from the aforementioned two cases of EBV-associated lymphoproliferative disorder ^88^. Thus, EBV-reactivation should be taken into account when using romidepsin to treat EBV-associated lymphoma.

Several romidepsin-based regimens are being evaluated. In the front-line setting, romidepsin plus CHOP was administered in 37 patients with PTCL ^65^. Although hematologic toxicities precluded the completion of the planned treatment in 18% of patients, the CR rate was 51%, the PR rate was 17%, and the OS rate at 30 months was 70.7%. Prospective trials with romidepsin plus CHOP (NCT01796002) or CHOEP-21 (NCT02223208) are ongoing.

In the salvage setting, romidepsin at 12 mg/m ^2^ (days 1, 8, and 15) with gemcitabine at 800 mg/m ^2^ (days 1 and 15) up to six cycles, followed by romidepsin maintenance (at 14 mg/m ^2^), was given to 20 patients with rrPTCL ^94^. The clinical outcomes of the combination were a little better than those with monotherapy; the CR rate and the ORR were 15% and 30%, respectively. Another phase I study of romidepsin, gemcitabine, dexamethasone, and cisplatin was carried out by a Canadian group ^95^, and of the 10 patients with PTCL, five responded. In addition, when romidepsin was added to ICE ^96^, the CR rate was 64% (9/14) and the ORR was 78% (11/14). However, grade 3–4 thrombocytopenia and neutropenia occurred in 95% and 84% of the cycles, respectively.

### Belinostat

Belinostat is a hydroxamic acid-derived, pan-HDAC inhibitor that demonstrates high affinity for the class I and II HDACs. In an early phase II trial, 24 patients with rrPTCL received belinostat, the ORR was 25% (6/24), and two patients experienced CR ^97^. In the phase II BELIEF (A Multicenter, Open Label Trial of Belinostat in Patients With Relapsed or Refractory Peripheral T-Cell Lymphoma) trial, 129 patients with rrPTCL were enrolled to receive belinostat at 1,000 mg/m ^2^ for five days every three weeks ^98^. Among 120 evaluable patients, 13 achieved CR (11%) and 18 achieved PR (15%), and the ORR was 26%. In patients with CR, the median DoR exceeded 29 months. A subtype analysis revealed that, among 22 patients with AITL, the ORR was 45% (four CRs and six PRs) ^99^. Most adverse events—nausea (42%), fatigue (37%), and pyrexia (35%)—were non-hematologic. Only 7% and 10% of patients experienced grade 3–4 thrombocytopenia and anemia, respectively.

These favorable hematologic toxicities provided momentum for initiating the next combination trial. In a phase I trial, belinostat at a dose of 1,000 mg/m ^2^ for five days every three weeks was combined with CHOP for six cycles in 23 patients with untreated PTCL ^100^. The combination was well tolerated; 18 patients (78%) completed six cycles. Among 21 evaluable patients, the ORR was 86% (n = 18), and most patients comprising the ORR were CR (n = 14, 67%).

### Chidamide

Chidamide is an orally administered benzamide class of HDAC that demonstrates selective inhibition of HDAC1, 2, 3, and 10 ^101^. In a phase II trial, 83 patients with rrPTCL took 30 mg of chidamide twice weekly ^102^. Among 79 evaluable patients, 11 CRs (14%) and 12 PRs (15%) were noted, and the median time to response was 1.4 months and the median DoR was 9.9 months. Grade 3–4 thrombocytopenia occurred in 22% of patients, and neutropenia occurred in 11% of patients. On the basis of these results, the Chinese Food and Drug Administration approved the use of chidamide to treat rrPTCL in 2014. Subsequently, large-scale real-world data were recently released ^103^. Chidamide monotherapy resulted in an ORR of 39% (100/256) and a CR rate of 9% (27/256). Chidamide-based combination therapies demonstrated the highest ORR (51%, 65/127) and CR rate (12%, 15/127). In the monotherapy group, grade 3–4 thrombocytopenia occurred in 10.2% of cases and neutropenia occurred in 6.2% of cases. The real-world data demonstrated clinical outcomes similar to those of the phase II trial. Several trials with chidamide-based combinations (NCT02809573, NCT02987244, NCT02856997, and NCT03023358) are ongoing.

### Other histone deacetylase inhibitors

Vorinostat is an orally administered benzamide HDAC inhibitor of class I–II. In a front-line setting, a combination of vorinostat plus CHOP demonstrated 79% and 81% of two-year PFS and OS rate, respectively ^104^. In a relapsed or refractory setting, a combination of vorinostat, lenalidomide, and dexamethasone (40 mg once daily) was explored ^105^, but the outcomes were not remarkable.

Panobinostat is an orally administered pan-HDAC inhibitor. In a phase II study ^106^, 25 patients with rrPTCL or ENKTL received panobinostat at a dose of 20 mg three times a week and bortezomib at a dose of 1.3 mg/m ^2^. The ORR was 43% (10/23), and the CR rate was 22% (5/23). Common treatment-related grade 3–4 toxicities were thrombocytopenia (68%), neutropenia (36%), and diarrhea (28%).

## Immunomodulatory drugs

Lenalidomide shows anti-lymphoma efficacy through immune modulation of the microenvironment and anti-proliferative and anti-angiogenic mechanisms ^107^. Two trials have investigated single-agent lenalidomide for 21 out of 28 days in patients with rrPTCL ^108, 109^. The CR rate ranged from 8% to 30%, and the ORR ranged from 22% to 30%. A substantial portion of patients (26–35%) experienced toxicities and this led to treatment discontinuation. Outcomes in patients with AITL, compared with other subtypes, were unique ^108^ and this suggests a role for the microenvironment in this subtype. In a phase I/II trial, a combination of romidepsine, lenalidomide, and carfilzomib was tried in patients with rrPTCL ^110^. In 16 evaluable patients, the CR rate was 31% (5/16) and the PR rate was 19% (3/16). Again, four out of five patients with AITL attained CR. A phase II study for untreated PTCL patients using lenalidomide plus romidepsin (NCT02232516) is ongoing.

Thalidomide, a prototype drug of this class, was evaluated for combination treatment. A combination of gemcitabine, cisplatin, prednisone, and thalidomide (GDPT) was compared with CHOP for patients with newly diagnosed PTCL ^111^. In total, 103 patients were randomly allocated into two groups; 52 received GDPT and 51 received CHOP. The PFS, the primary end-point of the study, was significantly better in the GDPT arm; the two-year PFS rates were 57% versus 35% ( *P* <0.05). Other outcomes, including the ORR (67% versus 49%, *P* = 0.046) and the two-year OS (71% versus 50%, *P* <0.05), were also favorable for the GDPT arm. Grade 3–4 myelosuppression occurred in 44% versus 41% of patients.

## Proteasome inhibitors

In a phase II trial, the single-agent bortezomib induced an ORR of 67% in 15 patients with rrCTCL ^112^. Because a chemosensitization effect was expected ^113^, the efficacy of bortezomib was explored in combination with CHOP in patients with newly diagnosed PTCL/ENKTL/CTCL ^114^. Of the 65 patients, 30 achieved CR (65%) and five other patients achieved PR. The ORR was 76% (35/46). When only three major subtypes of PTCLs (PTCL-NOS, AITL, and ALCL) were analyzed, the CR rate was 73% and the ORR was 87%. However, owing to frequent relapse after remission, the three-year OS and PFS rates were 47% and 35%, respectively. In another trial by the French group, the combination of bortezomib plus intensified CHOP-like regimen (ACVBP) was studied in 57 patients with newly diagnosed PTCL ^115^. The outcomes did not appear to be higher than chemotherapy alone, as the CR rate was 45%.

A newer, irreversible proteasome inhibitor (carfilzomib) is currently under investigation in patients with PTCL (NCT01336920 and NCT03141203). Ixazomib, an oral proteasome inhibitor, is being assessed in a phase 2 study (NCT02158975).

## Inhibitors of PI3K/mTOR pathways

PI3Ks transduce signals from various growth factors and cytokines into intracellular molecules by generating phospholipids, which then activate downstream effectors such as AKT or mTOR ^116^.

Duvelisib, a dual inhibitor of PI3Kγ and δ, was studied in a phase 1 trial in patients with hematologic malignancies ^117^. Among 15 evaluable patients with rrPTCL, the ORR was 47% (7/15, two CRs and five PRs) and the median OS was 36.4 weeks. A study of duvelisib with either romidepsin or bortezomib in T-cell lymphoma is under way (NCT02783625). Another PI3K δ/γ inhibitor, RP6530, was investigated in patients with relapsed or refractory T-cell lymphoma ^118^. Among 14 evaluable patients, one CR (7%) and four PRs (29%) were noted. Copanlisib, a pan-class I inhibitor, was administered at a dose of 0.8 mg/kg on days 1, 8, and 15 of a 28-day cycle in a phase II study in which 17 patients with PTCL were included, and the ORR was 21.4% ^119^. A phase I/II study for combination of copanlisib plus gemcitabine in rrPTCL is ready to begin (NCT03052933).

Given that a substantial portion of patients with PTCL show phospho-AKT overexpression, which confers a poor prognosis ^120^, AKT can be a reasonable target. In a phase II trial with MK2206, however, a frustrating result was observed; none of the three patients with PTCL responded ^121^.

Activation of mTOR induces cell growth and survival in cancer, and especially in lymphoma, Myc activity is known to depend on the mTOR pathway ^122^. In a phase II study of 30 patients with untreated PTCL who received everolimus plus CHOP ^123^, CR was observed in 17 (57%) and PR was observed in 10 (33%). Despite these favorable tumor responses, frequent relapses were noted as the two-year PFS rate was 33%. Single-agent everolimus in 16 patients with relapsed or refractory T-cell lymphoma demonstrated an ORR of 44% and a median DoR of 8.5 months ^124^.

## Alternative agents

### Bendamustine

Bendamustine, which contains the structures of both alkylating agents and purine analogs, is one of the standard agents in indolent B-cell lymphoma. In the phase II BENTLY (Bendamustine in Patients With Refractory or Relapsed T-cell Lymphoma) trial, 60 patients with refractory or relapsed T-cell lymphoma received bendamustine at a dose of 120 mg/m ^2^ for two consecutive days every three weeks for up to six cycles ^125^. With the majority of patients having AITL and PTCL-NOS (91%), the ORR was 50% and the median DoR was 3.5 months. Major grade 3–4 toxicities were neutropenia (30%), thrombocytopenia (24%), and infection (20%). In the phase II BENCART (Bendamustine, Carboplatin and Dexamethasone for Refractory or Relapsed Peripheral T-Cell Lymphoma) trial, 30 patients with rrPTCL received a combination of bendamustine, carboplatin, and dexamethasone to proceed to ASCT ^126^. Among 28 evaluable patients, eight CRs (30%) and seven PRs (25%) were observed. The median PFS was 4.8 months.

### Aurora-A kinase inhibitors

Aurora-A is a mitotic kinase overexpressed in several subtypes of PTCL ^127^. Alisertib, a small-molecule inhibitor of aurora-A kinase, has demonstrated favorable anti-tumor efficacy against rrPTCL in two phase II trials ^128, 129^. Given the promising results, a phase III LUMIERE (Alisertib or Investigator's Choice in Patients With Relapsed/Refractory Peripheral T-Cell Lymphoma) trial was performed in patients with rrPTCL to compare the efficacy of alisertib versus the investigator’s choice, including pralatrexate, romidepsin, or gemcitabine ^130^. With a planned accrual of 271 patients, interim analyses were carried out after 238 patients were recruited. ORRs, the primary end-points of the study, were 33% for alisertib and 43% for the investigator’s choice ^130^. In addition, no benefit was observed across the safety profiles. With these results, the trial was prematurely terminated.

### Tipifarnib

Tipifarnib is an orally administered nonpeptidomimetic farnesyl transferase inhibitor. In a phase II trial, 93 patients, including 16 patients with T-cell lymphoma, received tipifarnib 300 mg twice daily for three out of four weeks ^131^. A higher response rate was observed in the T-cell/Hodgkin’s lymphoma cohort (31%) compared with the aggressive (17%) or indolent (7%) B-cell lymphoma cohorts. Among eight patients with PTCL-NOS, three achieved CR and one achieved PR. Based on these promising results, a phase II trial was carried out solely on patients with rrPTCL, and the preliminary results were recently reported ^132^. A total of 18 patients received tipifarnib at a dose of 600 mg twice daily on days 1–7 and 15–21 in 28-day cycles, which demonstrated three PRs (17%) at the cost of grade 3–4 neutropenia (83%) and thrombocytopenia (61%).

### CPI-613

CPI-613 is a novel lipoate derivative that inhibits mitochondrial metabolism in cancer cells ^133^. In a phase I trial in patients with hematologic malignancies, the maximum tolerated dose was determined to be 2,940 mg/m ^2^, a major toxicity was renal failure ^134^. In another phase I trial in patients with relapsed or refractory T-cell lymphoma, bendamustine plus CPI-613 was administered ^135^. Out of five evaluable patients, three CRs and one PR were observed.

## Conclusions

Despite recent progress, there are hurdles to overcome in managing patients with PTCL, such as the poorly understood role of certain molecular features. Given the insufficient clinical outcomes out of the current standard of care, there are still unmet needs for the novel therapy.

Owing to the introduction of novel therapeutic agents however, recent outcomes are worthy of attention. Tailored clinical approaches regarding what drugs to initiate, when to consolidate patients, and how to best salvage patients require further investigation, including prospective trials.
